# Exposure Characteristics of Hantavirus Pulmonary Syndrome Patients, United States, 1993–2015

**DOI:** 10.3201/eid2305.161770

**Published:** 2017-05

**Authors:** Annabelle de St. Maurice, Elizabeth Ervin, Mare Schumacher, Hayley Yaglom, Elizabeth VinHatton, Sandra Melman, Ken Komatsu, Jennifer House, Dallin Peterson, Danielle Buttke, Alison Ryan, Del Yazzie, Craig Manning, Paul Ettestad, Pierre Rollin, Barbara Knust

**Affiliations:** Centers for Disease Control and Prevention, Atlanta, Georgia, USA (A. de St. Maurice, E. Ervin, C. Manning, P. Rollin, B. Knust);; Coconino County Public Health Services District, Flagstaff, Arizona, USA (M. Schumacher);; Arizona Department of Health Services, Phoenix, Arizona, USA (H. Yaglom, K. Komatsu);; New Mexico Department of Health, Santa Fe, New Mexico, USA (E. VinHatton, S. Melman, P. Ettestad);; Colorado Department of Health, Denver, Colorado, USA (J. House);; Utah Department of Health, Salt Lake City, Utah, USA (D. Peterson);; National Park Service, Fort Collins, Colorado, USA (D. Buttke);; Navajo Department of Health, Window Rock, Arizona, USA (A. Ryan, D. Yazzie)

**Keywords:** hantavirus, hantavirus pulmonary syndrome, occupation, viruses, United States, zoonoses

## Abstract

Those at highest risk are persons in occupations with potential for rodent exposure and American Indian women 40­–64 years of age.

Hantaviruses are negative-sense, single-stranded RNA viruses in the family *Bunyaviridae* (*1*). Hantavirus infections in humans are associated with several disease syndromes, including hemorrhagic fever with renal syndrome and hantavirus pulmonary syndrome (HPS; also known as hantavirus cardiopulmonary syndrome) (*2*,*3*). Although hantavirus infections had long been recognized in Asia and Europe, a 1993 outbreak of severe pulmonary disease in the Four Corners area of the United States (i.e., Utah, New Mexico, Arizona, Colorado) led to the discovery of Sin Nombre virus, the leading cause of HPS in the United States (*1*,*4*). In 1995, HPS became a nationally notifiable disease; the Viral Special Pathogens Branch (Division of High-Consequence Pathogens and Pathology, National Center for Emerging and Zoonotic Infectious Diseases) at the Centers for Disease Control and Prevention (CDC) maintains an HPS surveillance system and registry of reported HPS cases in the United States (*5*,*6*).

In the United States, most HPS cases are caused by Sin Nombre virus, for which the North American deer mouse (*Peromyscus maniculatus)* serves as reservoir (*7*). Other New World hantaviruses that cause human disease in the United States include New York and Monongahela viruses, transmitted by the North American deer mouse and white-footed deer mouse (*Peromyscus leucopus*); Black Creek Canal virus, transmitted by the hispid cotton rat (*Sigmodon hispidus*); and Bayou virus, transmitted by the marsh rice rat (*Oryzomys palustris*). Infected rodents excrete virus in their saliva, urine, and feces; inhalation of virus in rodent-infested areas is thought to be the primary mode of transmission to humans, although direct inoculation through a rodent bite is possible (*8*–*10*). Previous case–control studies have identified risk factors for HPS, such as having high rodent densities in the home; handling rodents; and performing cleaning activities, such as sweeping, in rodent-infested areas (*9*,*10*). Other factors that may precipitate exposure to hantaviruses include occupational and recreational activities, such as working outdoors or camping (*9*,*11*).

We sought to further describe demographics of HPS case-patients and possible occupational and environmental exposures associated with HPS. We examined surveillance data collected by the national HPS surveillance system.

## Methods

Since 1993, as part of national surveillance activities, state and local health departments have provided CDC with standardized clinical and exposure information for all laboratory-confirmed HPS cases (*12*). To be included as an HPS case-patient, patients were required to have no other cause of illness and to have an acute febrile illness with unexplained acute respiratory distress syndrome or evidence of interstitial pulmonary infiltrates on chest radiograph or to have an unexplained respiratory illness that resulted in death and an autopsy finding compatible with noncardiogenic pulmonary edema (*13*). In addition, all case-patients had laboratory confirmation of infection by either hantavirus-specific serologic testing (IgM and IgG) or reverse transcription PCR.

State and local health departments recorded all case-patient data on standardized surveillance case report forms, which asked closed-ended questions about case-patient demographics and open-ended questions about exposure (location and activities) and occupation (Technical Appendix Figure). For 158 cases from 1993–1999, investigators used a structured questionnaire to interview case-patients or family member proxies as part of routine surveillance; the methods used to collect these data and preliminary summary data have been published (*9*). The data gathered from the questionnaires were used to supplement the data included in the surveillance case report forms for the early case-patients. These data contained more detailed, systematically collected information about exposures, including specific questions about rodent exposure at home, in a recreational setting, and in the workplace.

On the basis of the free-text descriptions of reported rodent exposures and their locations, we classified rodent exposures as occurring at the case-patient’s home, at work, or in a recreational setting. Case-patients could be classified by >1 potential exposure setting. We also noted whether reported rodent exposure occurred in cars, trailers, or mobile homes and whether reported exposure included cleaning a rodent-infested area, regardless of the setting in which the exposure probably occurred. Among case-patients with a reported occupation, we created 2 categories: occupations for which direct or indirect contact with rodents was likely (i.e., outdoor activities or cleaning) and occupations for which such contact was unlikely (i.e., primarily indoors and office based). We subclassified occupations with opportunities for rodent contact as forestry/outdoor recreation, agriculture/ranching, construction/landscaping, professional cleaning, animal handling (e.g., wildlife biologist or exterminator), or oil field work. We defined the eastern United States as states east of the Mississippi River. We compared differences in frequency distribution between groups by using the Pearson χ^2^ for categorical variables and differences in means for all continuous variables by using the Student *t* test with unequal variance. We considered results statistically significant if the p value was <0.05.

## Results

During 1993–2015, a total of 662 laboratory-confirmed HPS case-patients were reported to CDC and included in the analysis. Of 651 case-patients for whom outcome information was recorded, 230 (35%) died; case-fatality rates did not vary by geographic region (p>0.05).

Race information was recorded for 648 case-patients. Most (78%) were white, although American Indians accounted for 18% of case-patients (Table 1). Most (89%) American Indian case-patients resided in the Four Corners area. American Indian case-patients were significantly younger than white case-patients (mean 34 vs. 39 years of age, respectively; *t* = 2.71, df = 164, p = 0.01), and the case-fatality rate among American Indian case-patients was significantly higher than that among white case-patients (46% vs. 33%, respectively; χ^2^ = 6.4, df = 1, p = 0.01). After stratification by age group, case-fatality rates were significantly higher among American Indian women 40–64 years of age than among white women of the same age group (Table 2).

**Table 1:** Demographics of laboratory-confirmed hantavirus pulmonary syndrome case-patients, United States, 1993–2015*

Case-patients, n = 662	No. (%)
Age, y	
<18	53 (8)
18–39	303 (46)
40–64	250 (38)
>65	48 (7)
Race, n = 648	
White	488 (78)
American Indian	113 (18)
Black	8 (1)
Asian/Pacific Islander	10 (2)
Other	1 (<1)
Male, n = 655	414 (63)
US Region, n = 662	
Eastern	27 (4)
Western	635 (96)
Not Hispanic, n = 525	404 (77)
Employment status	
Not reported	212 (32)
Unemployed	29 (4)
Retired	28 (4)
Student	39 (6)
Employed with reported occupation	354 (54)
Reported occupations, n = 354	
No frequent rodent exposure	190 (54)
Potential frequent rodent exposure	164 (46)
Agriculture/ranching†	80 (49)
Construction/landscaping‡	43(26)
Forestry/parks/outdoor recreation§	14 (9)
Cleaning¶	12 (7)
Oil field#	9 (5)
Animal work**	6 (4)

*Median patient age (interquartile range) 37 (26–50) years. †Farmer, rancher, rodeo worker, feedlot rider, dairy manager, bovine hoof trimming specialist, hay transporter. ‡Masonry, roofer, horticulturalist, electrician, building inspector, appliance repair, field laborer, and surveyor. §Conservation worker, rafting outfitter, fisheries technician, outdoor guide, outdoor researcher with no direct animal contact.  ¶Janitor and carpet cleaner.  #Well digger, oil field worker. **Small mammal researcher, exterminator.

**Table 2:** Hantavirus pulmonary syndrome deaths, stratified by patient race, sex, and age group, United States, 1993–2015

Patient age, y		American Indian male, no. (%)	White male, no. (%)	χ^2^	p value*	American Indian female, no. (%)	White female, no. (%)	χ^2^	p value*
<18		3 (38)	6 (26)	0.38	0.54	3 (30)	5 (56)	1.3	0.26
18–39		13 (42)	54 (38)	0.14	0.71	12 (50)	28 (37)	1.2	0.27
40–64		5 (31)	35 (29)	0.037	0.85	11 (69)	21 (30)	8.1	0.004
>64		1 (33)	6 (29)	0.029	0.87	1 (100)	3 (23)	2.7	0.10

*df = 1.

Rodent exposure was reported for 319 persons. We classified rodent exposure settings as being in the home, in a recreational setting, or at work (Table 3). Home exposure was most frequent in the eastern and western United States; however, home exposure was significantly more common among case-patients residing in the western United States (Table 3). Rodent exposure in a recreational setting was more common among case-patients residing in the eastern United States. Rodent exposures in cars, trailers, or mobile homes were reported for 49 (7%) case-patients. A history of cleaning a probable rodent-infested area (e.g., crawl spaces or outbuildings) was reported for 114 (17%) case-patients. The proportion of home exposures was greater for American Indian than for white case-patients (Table 4).

**Table 3:** Frequency of recorded rodent exposure types by US region of hantavirus pulmonary syndrome case-patients, United States, 1993–2015

Exposure	All case-patients, no. (%), n = 319	Western region, no. (%), n = 302	Eastern region, no. (%), n = 17	χ^2^	p value*
Home	228 (71)	220 (73)	8 (47)	5.2	0.022
Occupational	102 (32)	96 (32)	6 (35)	0.091	0.76
Recreational	78 (24)	70 (23)	8 (47)	5.0	0.026

*df = 1.

**Table 4:** Frequency of recorded rodent exposures by race for hantavirus pulmonary syndrome case-patients, United States, 1993–2015

Exposure	White, no. (%), n = 255	American Indian, no. (%), n = 43	χ^2^	p value*
Home	181 (71)	37 (86)	4.3	0.039
Occupational	90 (35)	7 (16)	6.1	0.014
Recreational	63 (25)	6 (14)	2.4	0.12

*df = 1.

Occupation status was reported for 450 (68%) case-patients, and a specific occupation was reported for 354. Those with occupations for which contact with rodents was deemed unlikely (e.g., teaching or clerical work) accounted for 54% of case-patients with a reported occupation (Table 1). Further analysis of the frequency of occupational exposure among 187 persons (28% of total case-patients) with both reported occupation and exposure (Table 5) indicated that those who worked in an occupation for which frequent rodent contact was possible were more likely to be occupationally exposed than those who worked in an occupation without the potential for frequent rodent exposure.

**Table 5:** Occupation risk and frequency of reported rodent exposure type for hantavirus pulmonary syndrome case-patients with specified occupation and exposure, United States, 1993–2015

Exposure	Occupation without frequent rodent exposure, n = 91	Occupation with potential frequent rodent exposure, n = 96	χ^2^	p value*
Home	67 (74)	60 (63)	2.7	0.10
Occupational	34 (37)	51 (53)	4.7	0.030
Recreational	22 (24)	15 (16)	2.2	0.14

*df = 1.

## Discussion 

Using exposure data for >600 case-patients reported by the national HPS surveillance system, we were able to define occupations and exposures that may contribute to increased risk of acquiring HPS; in this regard, our findings are consistent with those of previous studies. Early surveillance data identified possible risk factors for acquiring hantavirus infection as cleaning or entering structures that had been previously closed or uninhabited for long periods (*8*). Our study demonstrated that a possible source of hantavirus exposure may be cleaning rodent-infested areas because 17% of case-patients had a recorded history of cleaning areas that may have been rodent infested. Zietz et al. demonstrated that HPS was more likely to develop in herders but that risk was not increased for ranchers, farmers, and construction workers; however, their study was limited by small numbers of case-patients and was restricted to the Four Corners region, where herders are relatively overrepresented among occupations with rodent-exposure risk (*9*). In addition, given the dry, dusty environment in the Four Corners region and the likelihood of inhaling infected matter, persons in this region may be increasingly exposed to infected dust. Previous serologic studies of persons with occupational risk for rodent exposure did not reveal many with serologic evidence of past infection (*14*–*17*). However, because HPS is rare (i.e., typically 20–40 cases are reported in the United States annually), serologic surveys may not accurately portray risk for exposure to hantavirus when incidence is very low. We identified 2 cases, in addition to 3 previously published case reports, of HPS in persons who were not wearing adequate personal protective equipment while trapping wild mice for field research studies (*18*,*19*) and for whom direct contact with rodents in an occupational setting may have contributed to their risk. Therefore, the use of staff training along with appropriate personal protective equipment in field research settings (*20*) should be emphasized.

We identified that persons with occupations with potential for frequent rodent exposure should be aware of the risks for hantavirus infection; these persons include those working in agriculture (e.g., farmers, ranchers, and temporary laborers), construction (e.g., electricians, carpenters and roofers), forestry/outdoor recreation, oil drilling, and the cleaning industry (e.g., janitors and house cleaners). Employers should continue to educate employees about hantavirus transmission, steps to take to reduce the risk of contracting hantavirus infection in the workplace, and signs and symptoms of hantavirus infection. Current examples of employee education programs include informational sessions for river rafters and power industry workers in Arizona and industrial hygiene workers in Colorado and prevention education for National Park Service, Bureau of Land Management, and mining industry employees in New Mexico. Online educational materials for employees with frequent rodent exposure can be found at the websites of California Department of Public Health and the National Park Service (Technical Appendix Table).

Educational efforts to reduce exposure risk in the home should be continued because 71% of case-patients with a specified exposure reported rodent exposure at home. During the 1993 Four Corners outbreak, a case–control study found a significant association between higher rodent densities in the home and HPS (*10*). Our study echoed earlier surveillance data that identified risk factors to be cleaning or inhabiting structures that had been previously closed or uninhabited, because many of these structures may be rodent infested (*8*). Typical domestic cleaning activities, such as sweeping and vacuuming, are presumed to increase risk by aerosolizing infectious excreta. When performed in a confined area with limited ventilation, these activities may expose persons to a sufficient inoculum of virus to lead to infection. Public education programs for prevention of HPS in the residential setting, such as the Seal Up, Trap Up, Clean Up campaign launched by the New Mexico Department of Health in 1994 and adopted nationally, emphasize safe cleaning methods (e.g., wet mopping) and exclusion and removal of rodents from the peridomestic environment (Technical Appendix Table) (*21*). Simple and relatively inexpensive rodent exclusion methods, including the application of expanding foam and wire mesh to eliminate points of entry into living spaces, effectively reduce rodent infestations in homes. Our study demonstrated that home exposure was more common among American Indian case-patients than among those of other racial/ethnic groups. Targeted rodent exclusion projects in American Indian communities have successfully decreased rodent intrusion (*22*). Support for environmental health efforts aimed at rodent exclusion should be continued in American Indian communities. More recently, Navajo Nation has worked closely with CDC on a variety of educational projects, including presentations to Navajo Department of Health and Indian Health Service clinicians, an interactive radio forum on hantavirus, development of radio public service announcements in the Navajo language, and workshops with students at Dine College (Tsaile, AZ) to develop health communication videos for the general public.

A recreational exposure was recorded for 78 (24%) case-patients, 10 of whom were exposed during the 2012 outbreak in Yosemite National Park (*11*). The National Park Service has increased its efforts to educate visitors through its website, park brochures, and posters (Technical Appendix Table). In some settings, the National Park Service encourages overnight visitors to read a brief statement about hantavirus and prevention methods. The National Park Service is in the process of developing a comprehensive smartphone application for visitors. This application will not only serve as a resource for general details about the parks but will also contain information about safety precautions and animalborne diseases in the park. Because recreational exposures were proportionally more frequent among case-patients residing in the eastern United States, clinicians (even those caring for patients in low-incidence states) should assess recent travel history in addition to rodent exposures in the home and at work and consider hantavirus as a possible cause of disease.

Over the past few decades, educational materials on HPS and hantavirus for general audiences have been developed by health departments and distributed through local jurisdictions. A variety of local efforts to increase hantavirus awareness exist, through traditional and nontraditional news sources. These interventions are relevant, particularly in the spring when hantavirus infection prevalence may be higher among North American deer mice (*23*,*24*) and when persons may be more likely to participate in cleaning or recreational activities that could increase risk for rodent exposure. In 2016, spring electric bills in a Colorado county were accompanied by letters containing hantavirus information. Arizona works collaboratively with local public health and environmental health agencies to share prevention messages with the public to minimize the risk for rodent exposure in recreation, occupation, and peridomestic settings. The Coconino County (AZ) Public Health Department also posts preventive messages on Facebook and Twitter.

States in which risk for HPS is high send seasonal Health Alert Network messages to public health staff and clinicians. The New Mexico Department of Health answers hantavirus-related questions through an all-hours phone line and informs the public of new cases and prevention techniques through statewide press releases. On a national level, CDC manages a Hantavirus Hotline, which the general public and providers can call with hantavirus-related questions (online Technical Appendix Table). CDC, New Mexico Department of Health, and clinicians from the University of New Mexico (Albuquerque, NM) have given educational seminars to healthcare providers through the University of New Mexico Project ECHO, which targets Indian Health Service clinicians, and through Clinician Outreach and Communication Activity calls, which target a wide range of clinical professionals (Technical Appendix Table).

It is useful not only to define settings where HPS risk is increased because of rodent exposure but also to define demographic risk factors for HPS and subsequent death. HPS disproportionately affects American Indians, who represent ≈2% of the US population (*25*) yet account for 18% of reported US HPS cases. Because 89% of American Indian HPS case-patients reside in the Four Corners region, where most HPS cases occur, the disproportionate number of American Indian case-patients may in part result from environmental factors that increase the risk of inhaling infected dust particles. Biological factors that may increase HPS risk among American Indians have not been identified. We found that American Indians with HPS were younger and that mortality rates were significantly higher than those among whites of the same age group, particularly among American Indian women 40–64 years of age. According to the 2010 US Census, the median age for American Indians and Alaskan Natives is 28.8 years, compared with the median age for white Americans of 38.4 years (*25*); therefore, the age difference in our study may be a result of overall differences in age distribution between American Indians and white Americans. Sex disparities in death from HPS, by age, have been noted both within and outside the United States but are poorly understood (*26*–*29*). Different mortality rates could result from hormonal effects on the immune response, concurrent medical conditions, or exposure type. Among Norway rats infected with Seoul virus, immune responses vary by sex; Th1 response is greater for males than females (*30*). Of note, male and female humans with acute hantavirus infection have similar Th1 and Th2 responses but different levels of other cytokines, including interleukin-9, fibroblast growth factor 2, granulocyte macrophage colony–stimulating factor, and interleukin-8 (*31*). To prevent more cases and improve outcomes, investigations of the health disparities observed for American Indians and the increased mortality rates observed for American Indian women should continue.

Although we did not systematically collect information on physical location of rodent exposure, 49 case-patients were exposed in a vehicle, trailer, or mobile home. More information is needed to better understand if manufactured housing and vehicles increase the risk for rodent infestation and hantavirus exposure because of their construction. A recent HPS outbreak among overnight visitors to Yosemite National Park led to an association between staying in a particular type of housing (i.e., tents with drywall interiors) and risk for HPS (*32*). These tents were noted to have evidence of active rodent infestation, holes in the canvas, and gaps between the tent and insulated wall, enabling rodent entry. National Park Service employees and migrant workers (*33*) may also reside in temporary on-site housing or use vehicles provided by their employers; therefore, employers should also be prudent about excluding rodents from these items.

Our findings have several limitations. Because of underreporting or misdiagnosis, we may not have captured all cases of HPS in the United States. Ethnicity and race data were missing from 7% and 26% of case report forms, respectively, because some states have only recently begun collecting that information. Because occupation and exposure history were collected by use of free-text responses, data for these variables were not collected systematically for all reported cases. Persons completing case report forms may have overreported occupations for those persons who are more likely to have been exposed to rodents at work, and the HPS-associated exposure could have occurred at another site not reported on the case report form. In addition, for case-patients who lived at their workplace (e.g., forestry, agriculture), it was difficult to distinguish where rodent exposure occurred. As a result of this analysis, we have modified our case report form to systematically capture more detailed information regarding type of exposure and setting.

Although HPS is rare in the United States, surveillance data suggest that persons in certain occupations and certain populations may be at increased risk for HPS because of potential for rodent exposure. Physicians should recognize HPS risk factors and consider HPS for patients with documented rodent exposure or who are at high risk for rodent exposure. Educational efforts and awareness focused on high-risk populations should continue so that persons can decrease their risk of acquiring HPS.

Technical Appendix Resources for hantavirus education; hantavirus pulmonary syndrome case report form.
