# Supplementary material for: Exposure Characteristics of Hantavirus Pulmonary Syndrome Patients, United States, 1993–2015
Source: Emerg Infect Dis. 2017 May;23(5):733–9. doi: 10.3201/eid2305.161770 (PMC5403056; doi:10.3201/eid2305.161770)
Supplement: Technical Appendix — Resources for hantavirus education; hantavirus pulmonary syndrome case report form. [file 16-1770-Techapp-s1.pdf]

Article DOI: http://dx.doi.org/10.3201/eid2305.161770

Exposure Characteristics of Hantavirus Pulmonary Syndrome Patients, United States, 1993–2015

Technical Appendix

Technical Appendix Table. Online and telephone resources for hantavirus education

Website source, types of information provided	URL(s)
Arizona Department of Health<br>Information about vector control workshops in the community<br>Hantavirus fact sheets<br>Links to CDC Web site about hantavirus	http://azdhs.gov/preparedness/epidemiology-disease-control/vector-borne-zoonotic-diseases/index.php
California Department of Health<br>Training videos about hantavirus, safe methods for cleaning up after rodents and rodent exclusion techniques<br>Hantavirus self-test<br>Work place posters<br>Brochures	http://www.cdph.ca.gov/HealthInfo/discond/Pages/HantavirusToolkit.aspx
Centers for Disease Control and Prevention<br>General information about hantavirus and HPS<br>Seal up, Trap Up, Clean up manual on preventing rodent infestation<br>Hantavirus Clinician Outreach and Communication Activity (COCA)	http://www.cdc.gov/hantavirus/, http://www.cdc.gov/hantavirus/hps/prevention.html, https://emergency.cdc.gov/coca/calls/2016/callinfo_063016.asp
Colorado Department of Health<br>Information about hantavirus prevention at home and in the workplace<br>Information about cleaning up after rodents<br>Access to case investigation resources for local public health agencies<br>Colorado HPS Annual Reports	https://www.colorado.gov/pacific/cdphe/hantavirus
National Park Service<br>General information about hantavirus, HPS, and prevention methods for workers and park visitors<br>General hantavirus information and rodent exclusion brochure targeted to museum workers	https://www.nps.gov/grte/planyourvisit/hps.htm, https://www.nps.gov/museum/publications/conserveogram/02-08.pdf
Navajo Department of Health<br>Public Service Announcement for Hantavirus	http://www.nndoh.org
New Mexico Department of Health<br>Information for the general public, home owners, public health officials, occupational workers at risk of exposure and physicians about HPS, hantavirus prevention, rodent proofing, case investigation, hantavirus surveillance, and caring for HPS cases	https://nmhealth.org/about/erd/ideb/zdp/hps/
Utah Department of Health<br>Information for the general public and public health departments about hantavirus, case investigation, and preventing rodent infestations	http://health.utah.gov/epi/diseases/hantavirus/

Form Approved OMB No. 0920-0009

Hantavirus Pulmonary Syndrome Case Report Form

Please return to: Centers for Disease Control and Prevention, Special Pathogens Branch
Ph: (404) 639-1510 Fax: (404) 639-1118 Email: dvd1spath@cdc.gov
Site: www.cdc.gov/ncidod/diseases/hanta/hps/noframes/phys/specimen/hlthdept.htm

Patient Identification

__	__	__	__	__	__	__	__

-FIPS- -YR-

Information below is required for identification and meaningful interpretation of laboratory diagnostic results. HPS may not be confirmed without compatible clinical and/or exposure data.

PATIENT INFORMATION

Last name:

First name: MI:

Age: Sex: Choose one

Street address:

City/town:

County:

State: ZIP:

PATIENT'S BACKGROUND and EXPOSURE INFORMATION

Occupation:

Ethnicity: Choose one

Race: ☐ American Indian/Alaska Native ☐ Asian ☐ Black or African American ☐ White ☐ Native Hawaiian/other Pacific Islander

History of rodent exposure in 6 weeks prior to onset of illness? Choose one

If yes, type of rodent? Choose one

Place of contact (town, county, state):

Notes:

TIMELINE

Date of onset of symptoms: ____________________ Patient hospitalized? Choose one Date of hospitalization: ____________________

CLINICAL INFORMATION

Fever > 101° F (38.3° C)? Choose one

Thrombocytopenia? (platelets <150,000mm) Choose one

Lowest platelet count measured: ____________________

Elevated hematocrit (Hct)? Choose one

Highest hematocrit measured: ____________________

Elevated creatinine? Choose one

Highest creatinine measured: ____________________

WBC total: ____________________
Total neutrophils: ____________________ %
Band neutrophils: ____________________ %
Lymphocytes: ____________________ %

CLINICAL INFORMATION

Supplemental oxygen required? Choose one

Was patient intubated? Choose one

CXR with unexplained bilateral interstitial infiltrates or suggestive of ARDS? Choose one

OUTCOME

Outcome of illness? Choose one

Date of death: ____________________

Autopsy performed? Choose one

SPECIMEN INFORMATION

Specimen acquisition date: ____________________

Type of specimen: ____________________

Has specimen been tested for hantavirus at a laboratory? Choose one

If yes, where? ____________________

Results (i.e., titer, OD): ____________________

Notes:

FOR STATE HEALTH DEPARTMENTS

State Health Department reporting case: ____________________ State/local ID number: ________ Date form completed: ____________________

Person completing Report: ____________________ Phone number: ____________________

Name of patient's physician: ____________________ Phone number: ____________________

Instructions: You must have internet access and an email address to submit this Form electronically. Upon hitting the 'Submit by Email' button, a PDF is created, attached to an email, which you should then send to the address which appears in the address header; you may also cc: others. Acknowledgement of receipt by CDC is not provided.

Submit by Email

Public reporting burden of this collection of information is estimated to average 20 minutes per response, including the time for reviewing instructions, searching existing data sources, gathering and maintaining the data needed, and completing and reviewing the collection of information. An agency may not conduct or sponsor, and a person is not required to respond to a collection of information unless it displays a currently valid OMB control number. Send comments regarding this burden estimate or any other aspect of this collection of information, including suggestions for reducing this burden to CDC/ATSDR Reports Clearance Officer; 1600 Clifton Road NE, MS D-74, Atlanta, Georgia 30333; ATTN: PRA (0920-0009).

Technical Appendix Figure. Hantavirus pulmonary syndrome case report form.
